# A Snapshot of the Genetic Diversity of *Salmonella* Enteritidis Population Involved in Human Infections in Romania Taken in the European Epidemiological Context

**DOI:** 10.3390/pathogens10111490

**Published:** 2021-11-16

**Authors:** Codruta-Romanita Usein, Mihaela Oprea, Adriana Simona Ciontea, Sorin Dinu, Daniela Cristea, Lavinia Cipriana Zota, Saara Kotila

**Affiliations:** 1 “Cantacuzino” National Military Medical Institute for Research and Development, 050096 Bucharest, Romania; ilesimona@yahoo.com (A.S.C.); sorindinu30@gmail.com (S.D.); cristea_dana01@yahoo.com (D.C.); 2 National Center for Surveillance and Control of Communicable Diseases, National Institute of Public Health, 050463 Bucharest, Romania; lavinia.zota@insp.gov.ro; 3 European Centre for Disease Prevention and Control, 16973 Solna, Sweden; saara.kotila@ecdc.europa.eu

**Keywords:** *Salmonella* Enteritidis, salmonellosis, antimicrobial resistance, MLVA, MLST, cgMLST

## Abstract

In the absence of consistent national molecular typing data to enhance the surveillance of *Salmonella* Enteritidis, it was considered useful to collect baseline information on the genetic diversity and antibiotic susceptibility of strains isolated in Romania between January 2016 and April 2020 and compare them to strains described in major international outbreaks of the same period. A collection of 245 clinical isolates were genotyped by a standardised multiple-locus variable-number of tandem repeats analysis (MLVA) 5-loci protocol and screened for antimicrobial resistance against 15 compounds. Twenty strains were further subjected to whole genome sequencing (WGS) and compared to epidemiologically relevant high-throughput sequencing data available in European databases. Twenty-seven MLVA genotypes were identified, of which three, commonly reported in Europe between 2016–2020, covered 72% of the collection. Antibiotic resistance was detected in 30% of the strains, with resistance to nalidixic acid and ciprofloxacin as the most common phenotype, and also associated with two prevalent MLVA clones. WGS-derived multilocus sequence typing (MLST) revealed a single sequence type (ST11) further resolved into 10 core-genome MLST complex types. The minimum spanning tree constructed from the cgMLST data clustered Romanian and international strains, which shared more than 95% of the core genes, revealing links with a contemporaneous multi-country outbreak. This study could be regarded as a forerunner to the advent of using this integrative approach in the public health practice at a national level and thus contribute to the concerted actions at a European level to stop outbreaks.

## 1. Introduction

In 2019, the data provided by the European Food Safety Authority (EFSA) and the European Centre for Disease Prevention and Control (ECDC) showed that gastrointestinal infections involving *Salmonella enterica* ranked second among foodborne zoonotic infections at a European Union (EU) level, with a total of 87,923 confirmed cases and 926 foodborne illness outbreaks, mostly generated by serotype Enteritidis [1].

From 2012 and up to end of 2019, ECDC has supported the development of a molecular typing-enhanced surveillance of food-borne infections through the Food- and Waterborne Diseases and Zoonoses (FWD) program, advocating the usefulness of internationally agreed typing methods and data-sharing via the Epidemic Intelligence Information System (EPIS)—now replaced by EpiPulse/The European Surveillance System (TESSy), in order to improve early detection of multi-country outbreaks and outbreak investigation support at the EU and European Economic Area (EEA) level. For *Salmonella*, the globally used molecular typing tools used for comparing human, animal and food/feed isolates are pulsed-field gel electrophoresis (PFGE), the generic technique for typing all serotypes and multiple-locus variable-number of tandem repeats analysis (MLVA), a serotype-specific approach [2]. Furthermore, the integration of whole genome sequencing (WGS)-based methods into the European public health practice is strengthening outbreak investigations at a national and international level [3]. Yet, differences in the scope and capacity of molecular typing between the countries and the limited sharing of the molecular typing data for EU surveillance remain issues which diminish the capacity to monitor the spread of microbial clones and detect cross-border outbreaks [4].

In Romania, notification of non-typhoidal salmonellosis is mandatory, but implementation of a consistent strategy on the molecular typing of *Salmonella* at a national laboratory level was neglected before 2013, when the national public health authorities issued the recommendation that 25% of the *Salmonella* strains isolated by the county public health laboratories should be genotyped by PFGE in a referral centre. In spite of the agreed legislation, the proportion of typed isolates was insufficient to support national-level surveillance and response activities, most likely due to the scarcity of regional funding. 

In the EU official statistics by country and year on zoonoses, Romania was listed with a proportion of salmonellosis cases ranging between 5.9–7.5% per year in 2015–2019 and very high proportions of domestic cases [1]. This retrospective investigation aimed to provide information on the genetic diversity and antibiotic susceptibility of *S*. Enteritidis strains isolated in the framework of salmonellosis surveillance in Romania in the recent years. It was conducted using typing protocols harmonised at EU-level, if available, to allow interpretation of the results in a broader European context, although these methods have not yet been implemented in the Romanian surveillance scheme. Along with developing the capacity for WGS, we wanted to investigate how many salmonellosis cases in Romania cluster together and were linked to EU-wide outbreaks and if the treatment options could be improved for those cases. 

## 2. Results

### 2.1. MLVA Typing

All strains were typeable by MLVA. The diversity of the five loci used to represent the genome of the *S*. Enteritidis strains was evaluated by both the number of alleles and Simpson’s DI. All the loci were polymorphic with between two to nine alleles per locus and the DIs were from 0.085 to 0.600. Specifically, the most diverse loci were SENTR5, followed by SENTR6, and SENTR4 generating seven, nine, and four alleles, respectively, and polymorphism index values of 0.600, 0.382, and 0.235. Loci SENTR7 and SE3 were the least diverse, displaying two alleles, but SE3 had a higher diversity index value (0.214) compared to SENTR7 (0.085). 

Overall, the 245 Romanian *S*. Enteritidis strains were resolved into 27 distinct MLVA genotypes, designated G1 to G27, reflecting a Simpson’s DI of 0.728 (CI = 0.673−0.783). There were 14 multiple-strain and 13 single-strain MLVA genotypes. The frequency of the shared MLVA genotypes differed within the sampled collection; only three of them exhibited a prevalence of more than 10%, while 11 occurred with rates between 0.8–6%. 

MLVA genotype G1, defined by the allele string 2-11-7-3-2, was the most prevalent, accounting for 49% of the strains. Observed each year from 2016 to 2020, G1 was assigned to strains originating from sporadic cases (72 strains) and outbreaks (48 strains/9 outbreaks) dispersed in 19 counties. Notably, this MLVA genotype was represented by at least 35% of the strains of each sampling year. MLVA genotype G2, with the allelic profile 2-10-7-3-2, was the second in prevalence, accounting for 12% of the strains. It was shared by sporadic strains (16 strains) and strains originated from outbreaks (14 strains/2 outbreaks) isolated between 2016 and 2019 across 11 counties. MLVA genotype G3, defined by the allelic profile 2-12-7-3-2, ranked third in prevalence (11%). It was assigned to strains isolated between 2017–2019 from nine counties, causing infections with no epidemiological links (17 strains) and outbreaks (10 strains/2 outbreaks). 

There were two more MLVA allelic profiles besides G2 and G3, which were distinguished from the prominent MLVA profile G1 by the number of the tandem repeat units at SENTR5 locus, 2-9-7-3-2 (named G6) and 2-13-7-3-2 (named G9), respectively. The first profile was shared by nine of 154 strains (6%) isolated from sporadic infections, which occurred between 2016 and 2019. All the data regarding the MLVA genotypes in association with the county distribution over the studied period are summarised in Table 1.

**Figure 1:**
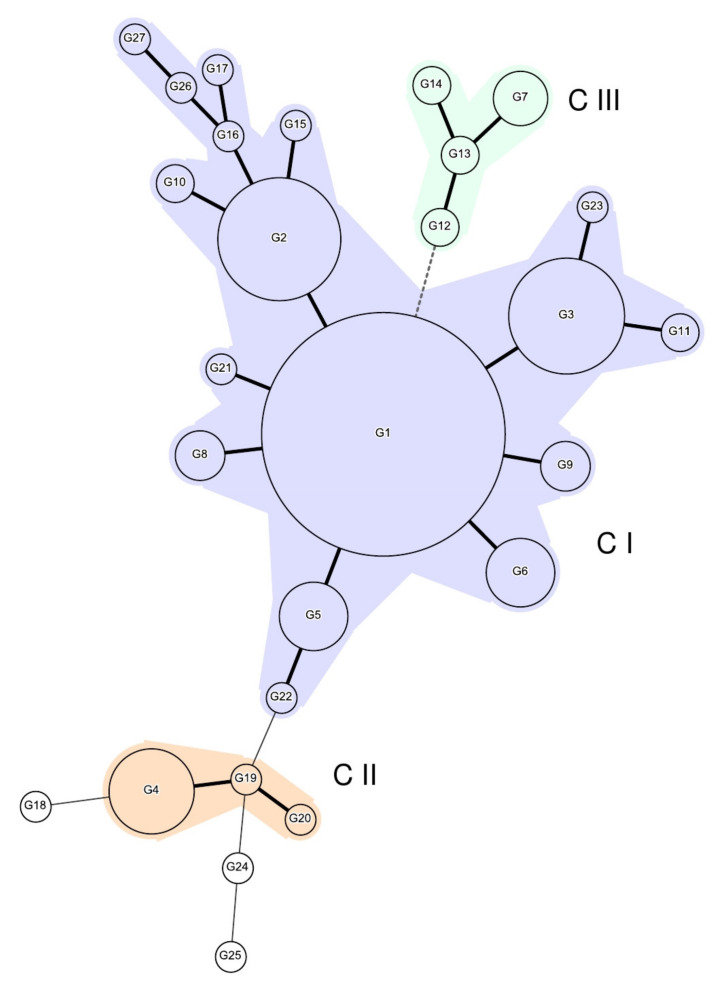
Minimum spanning tree showing the clonal relationship between the 245 *S*. Enteritidis strains genotyped by MLVA. The diagram includes the 27 distinct MLVA profiles, represented as circles, with size proportionate to the number of isolates (the smaller the node, the fewer the number of isolates in the MLVA profile), numbered from G1 to G27. MLVA profiles are connected by branches, and the type of the branch indicates the number of MLVA loci differences between the connected MLVA profiles. The thick solid lines connect MLVA profiles that differ by one locus, the thin solid lines connect MLVA profiles that have two loci difference, and the dotted line connect MLVA profiles that have three loci difference. MLVA clusters (CI-CIII) were defined by using one allele difference between two neighbouring MLVA genotypes as criterion.

MST analysis showed three MLVA clusters designated by Roman numerals (Figure 1).

Cluster CI was the largest comprising 16 MLVA genotypes (G1-G3, G5, G6, G8-G11, G15-G17, G21, G22, G26, and G27) assigned to 87% of the strains originating from both sporadic cases of infections and outbreaks. Within this cluster, G1, with its seven variants that displayed one allele difference at the most variable loci SENTR5 or SENTR6, grouped most of the strains. Cluster CII, delineated at double-locus variant level, consisted of three MLVA genotypes (G4, G19, and G20) assigned to strains originating mostly from the same outbreak. Cluster CIII, defined at three-locus variant level, comprised four MLVA genotypes (G7 and G12- G14) assigned to both sporadic strains and outbreak-related strains which was distinguished clearly from the other genotypes by the allele displayed at the most stable locus, SENTR7. Three genotypes (G18, G24, and G25) were singletons.

The 16 outbreaks investigated in this study were associated with six MLVA genotypes and only one of these MLVA types was not also assigned to at least one sporadic strain. Generally, within an outbreak the strains displayed identical MLVA genotypes, which confirmed the epidemiological links. Yet, strains that differed from the main MLVA genotype were also observed within the same outbreak (i.e., DB/2017, VS/2018, and MM/2019_1). Specifically, one strain differed from the rest across the human isolates associated with the outbreaks DB/2017 (G2 versus G8) and VS/2018 (G19 versus G4), respectively, whereas the food isolate displayed a different MLVA type than the human strains (G1 versus G7) linked to the MM/2019_1 outbreak.

Nine outbreaks which occurred over the entire observational period were associated with the prominent MLVA genotype G1 (AB/2020, B/2018, HR/2017, HR/2018, IS/2016, IS/2017_2, IS/2017_3, IS/2018, and MM/2019_2) and four outbreaks to its related variants G2 (IS/2019 and MM/2018), G3 (IS/2017_1 and PH/2018), and G8 (DB/2017), respectively. The strains originated from two more outbreaks from the years 2018 and 2019 and were attributed to the more distant MLVA genotypes G4 (VS/2018) and G7 (MM/2019_1), respectively.

### 2.2. WGS-Based Typing

Twenty *S*. Enteritidis strains, representative for MLVA genotypes G1 (five strains), G2 (nine strains), and G6 (six strains), were further typed at a whole genome resolution. They covered each year of the studied period and displayed closely related MLVA genotypes that have predominated in the collection or associated with important international outbreaks. In silico MLST assigned all to the sequence type ST11 while cgMLST separated them into 10 cgMLST Complex Types (CTs). The MLVA genotype G2 subset consisted of strains from a single outbreak, which occurred in 2018 (MM/2018 outbreak) and was assigned the same CT (CT4068). Within the MLVA genotype G1 group, each strain was assigned a distinctive CT (CT373, CT431, CT2058, CT6507, and CT6731) while the six strains representative for the MLVA genotype G6 were attributed four CTs (CT371, CT384, CT387, and CT4050).

**Figure 2:**
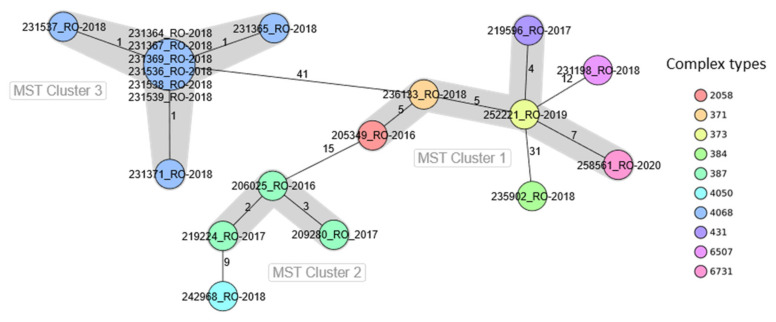
Minimum spanning tree based on core-genome multilocus sequence type allelic profiles for Romanian *S.* Enteritidis strains (*n* = 20) compared in this study with pairwise ignoring missing values. The analysis and tree were obtained with the public Ridom–SeqSphere+-integrated *S. enterica* cgMLST scheme of 3002 target loci. On the tree, allele distances between samples are indicated. Clusters of samples with maximum seven alleles distance are shaded in grey.

The MST generated from the cgMLST data grouped 17 strains in three clusters designated by Arabic numerals and left three strains as outliers because they exceeded the cluster distance threshold of seven alleles (Figure 2).

The most diverse cluster (Cluster 1) comprised strains assigned to different MLVA genotypes, namely G1 strains linked to sporadic cases (205349_RO_2016 and 252221_RO_2019) and two outbreaks (219596_RO_2017 from IS/2017_3 and 258561_RO_2020 from AB/2020) plus one G6 sporadic strain (236133_RO_2018). These strains had not known epidemiological relationships and were collected in different years from four non-neighbouring counties. The cluster outliers were one strain with the MLVA genotype G1 and CT6507 profile (231198_RO_2018) which originated in the IS/2018 outbreak and one MLVA G6 strain assigned to CT 2058 (235902_RO_2018) isolated from a sporadic infection. Cluster 2 held three sporadic strains, which shared the MLVA genotype G6 and CT387 (206025_RO_2016, 209280_RO_2017, and 219224_RO_2017), collected in consecutive years from different counties. The outlier strain (242968_RO_2018) with nine allele differences had the cluster MLVA genotype but a different cgMLST profile (CT4050). The most distinct and homogeneous cluster (Cluster 3) was built up from within outbreak strains, which displayed 0 to 1 allelic difference among them.

Apart from the gene-by-gene approach, the SNP analysis performed showed distances between 0 to 115 SNPs (average 66.14) among the genomes of these 20 *S*. Enteritidis strains. Specifically, there were 1–16 SNPs differences (average 6.5) between the outbreak-related strains, whereas the unrelated strains representative for MLVA genotypes G1 and G6 differed by 10–30 SNPs (average 19.2) and 8–104 (average 52.33), respectively.

**Figure 3:**
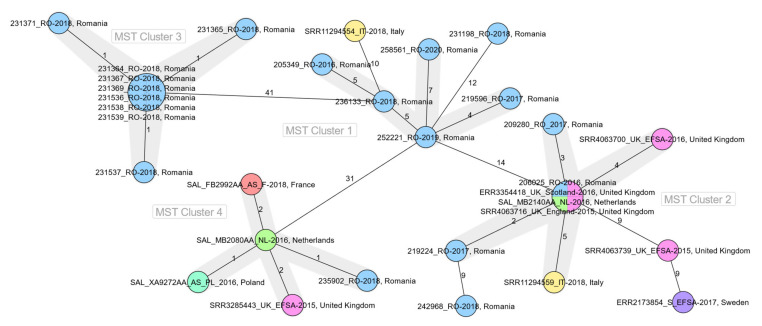
Minimum spanning tree based on core-genome multilocus sequence type allelic profiles for Romanian and other European *Salmonella* Enteritidis strains (*n* = 32). The analysis and tree were obtained with the public Ridom-SeqSphere+-integrated *S. enterica* cgMLST scheme of 3002 target loci. On the tree, allele distances between samples are indicated. The different colours are related to the countries of strains’ origin. Clusters of samples with a of maximum seven alleles distance are shaded in grey.

Genomic comparison revealed that four of the Romanian strains that shared the MLVA genotype G6 (206025_RO_2016, 209280_RO_2017, 219224_RO_2017, and 235902_RO_2018) were closely related with strains isolated in other European countries (between 0 to 5 differing alleles) although they were previously considered as sporadic cases. On the MST constructed with Romanian and other European sequence data, these strains were distributed in two of the four clusters delineated (Cluster 2 and Cluster 4), being grouped with strains from Italy, France, the Netherlands, Poland, and the United Kingdom, collected in 2015, 2016, and 2018 and all linked to a multi-country outbreak (Figure 3) [5].

In 19 of the 20 assemblies, 95% or more of the core loci were detected. The comparison against sequences available in TESSy confirmed the microbiological link to a multi-country outbreak for the four isolates mentioned in the previous paragraph, although for 209280_RO_2017 only 94.8% of the core loci were found. Furthermore, three additional isolates had matches from other European countries within six core genome alleles, two of them previously considered as sporadic and one related to a national outbreak (IS/2017_3).

### 2.3. In Vitro and In Silico Antibiotic Resistance in Relationship with the MLVA Type

Overall, 173 strains (71%) were pan-susceptible and 72 strains (29%) expressed various resistance rates to nalidixic acid (68 strains), ciprofloxacin (66 strains), pefloxacin (66 strains), sulphonamides (three strains), ampicillin (two strains), and streptomycin (one strain). Resistant strains were assigned 11 MLVA genotypes and genotypes G1 and G3 covered the majority of the strains resistant to ciprofloxacin and/or nalidixic acid (59 strains).

ResFinder tool detected an aminoglycoside acetyltransferase *aac(6′)*-*I* type gene with 96.35% identity with *aac(6′)*-*Iaa* in all the sequenced strains, although none of them expressed phenotypic resistance to the aminoglycosides used in this study. NCBI BLASTx revealed a six amino acid residues difference with the enzyme encoded by *aac(6′)-Iaa* (GenBank accession number NC_003197) and a difference of four residues with *aac(6′)-Iy* (GenBank accession number AF144880). Three strains with nalidixic acid resistance and low-level ciprofloxacin resistance (MIC 0.09–0.125 mg/L) carried only one *gyrA* mutation at codon 87, resulting in the substitution of aspartic acid with tyrosine (D87Y).

## 3. Discussion

When the ECDC and EFSA released an updated assessment of the public health risk associated with a prolonged international outbreak of *S*. Enteritidis infections linked to eggs, the conclusion was that the outbreak, which began in 2016, was ongoing and the source of contamination had not been eliminated [5]. Consequently, the public health and food safety authorities of the Member States were requested to continue sharing relevant epidemiological information along with WGS/MLVA results on human, food, and veterinary isolates in order to support trace-back investigations. Until Romania develops their typing/WGS capacity and integrates it as part of the national and EU-level surveillance of *Salmonella* infections, we considered it useful to pilot these approaches on a laboratory collection encompassing strains obtained through a national surveillance program of human gastrointestinal infections collected in the period coinciding with the international outbreak.

The Romanian *S*. Enteritidis collection proved to include many strains displaying indistinguishable or closely related MLVA profiles that differed by no more than one locus. The top most frequently identified MLVA profiles, 2-11-7-3-2 (G1), 2-10-7-3-2 (G2), and 2-12-7-3-2 (G3), highly related considering the minor differences in the most polymorphic locus SENTR5, accounted for a large proportion of the sampled population (73%). These were most likely the clones with importance from the public health perspective, at least since 2016, as they were present in more than two-thirds of the strains from nationally distributed sporadic cases and were associated with most of the community outbreaks investigated in this study. However, it appeared that the MLVA profile 2-11-7-3-2 was the most successfully spreading clone in Romania, as the corresponding strains had the broadest time period and geography of isolation and the highest frequency each sampling year throughout the observational period. Furthermore, antimicrobial susceptibility results provided evidence that a subpopulation of this major MLVA clone had acquired resistance to ciprofloxacin, a growing concern at the global level, which requires not only monitoring in view of the importance of fluoroquinolones in the treatment of severe enteric infections, but also prioritizes research for developing an alternative antimicrobial [6,7]. Official statistics at a European level indicated Romania as a country with statistically increasing trends in the resistance of *S*. Enteritidis to ciprofloxacin/nalidixic acid between 2013-2018 [8]. The finding of co-resistance to nalidixic acid and ciprofloxacin, as the most common antibiotic-resistant profile of the bacterial collection studied, could reflect this. Unfortunately, the scarcity of Romanian studies on the fluoroquinolone resistance of *Salmonella* strains of human origin and the wide variations between the susceptibility results reported by the studies of *Salmonella* in animals and animal products [9], make it difficult to seize the true public health significance of the fluoroquinolone-resistant *Salmonella* in the country and call for further monitoring of the presence and spread of fluoroquinolone resistance based on rigorous laboratory protocols. It is also worth noting that the integration of the WGS-inferred information allowed us to notice that fluoroquinolone-resistant strains phenotypically susceptible to aminoglycosides carried a *aac(6′)-I*-type gene whose predicted product differed from the aminoglycoside-acetylating enzyme AAC(6′)-Iy by only four amino acids. It is known that that *aac(6′)-Iy* is a usually silent chromosomal gene specific for *Salmonella* genus whose expression depends on molecular rearrangements occurring in its genomic environment [10].

The MLVA data provided insight into the epidemiology of *S*. Enteritidis by showing that the genotypes with most importance in human disease in Romania also had high epidemiological relevance in other European countries, as revealed by their presence among the 10 most commonly reported MLVA profiles at the EU/EEA level in 2016–2020 (unpublished data). Furthermore, according to their MLVA profiles, almost 9% of the Romanian *S.* Enteritidis human strains tested in this study qualified as causes of historical-probable and probable cases associated with the multistate EU outbreak caused by Polish eggs [5]. We partially addressed this issue by increasing the typing resolution to the WGS level in agreement with the European recommendations for the outbreak investigation of foodborne pathogens [11]. We tried to counterbalance the lack of bioinformatics expertise and the known difficulties of confidently delineating related and unrelated strains in the absence of a consensus for defining the SNP/allele threshold by using commercial software and an open-source SNP calling pipeline with default parameter settings for the WGS data analysis. It was apparent that the WGS-derived typing data were more useful for categorising the strains as related or unrelated, despite the indistinguishability by MLVA. In silico MLST showed that all sequenced strains belonged to ST11, the most common lineage of *S*. Enteritidis population [12]. cgMLST, which is considered of particular utility to distinguish very closely related strains of *S*. Enteritidis [13], provided evidence of possible cross-border transmission of strains commonly circulating at the European level and Romanian cases being likely linked to strains commonly circulating in other EU/EEA countries.

Due to the lack of resources, not all the strains with national and international epidemiological relevance could be sequenced and compared; this was one of the limitations of the study. Other limitations were the convenience strain sample, which may have represented a population that was not balanced, thus reducing generalizability and the minimal associated epidemiologic data limiting comparisons and unequivocal conclusions. Nevertheless, this study generated the first MLVA and WGS data which provided insights into the *S*. Enteritidis population structure documenting the presence of strains with genetic similarities spanning multiple years and counties in Romania and indicating connections to international outbreaks. Its results could be regarded as a forerunner to the advent of using this integrated approach in the public health practice as routine so we can have an up-to-date overview of the sources and of the spatial and temporal spreading of the autochthonous cases and also contribute to the concerted actions at the EU level to stop outbreak situations.

## 4. Methods

### 4.1. Bacterial Strains

Romania consists of 41 counties, along with the municipality of Bucharest (corresponding to 42 NUTS 3 units), each with their own public health laboratory responsible for the biochemical identification and preliminary serogrouping of *Salmonella* isolates collected from the clinical laboratories. Between January 2016 and April 2020, a total of 753 presumptive *Salmonella* isolates, the vast majority of them from human patients, were submitted by 28 of these laboratories to the Laboratory of Bacterial Enteric Infections (*Salmonella* National Reference Laboratory) of the “Cantacuzino” National Medical-Military Institute of Research and Development for confirmation and full serotyping. The data from the laboratory records showed that 441 of these strains were identified as *S*. Enteritidis (unpublished data). In this study, to include a variety of different *S*. Enteritidis, we selected 245 strains collected from 23 counties, which comprised 154 strains without known epidemiological relationship, therefore considering sporadic strains, and 91 strains with notification of outbreak origin. The latter subset was derived from 16 outbreaks (2–15 strains/outbreak) which were assigned a laboratory-specific nomenclature using the abbreviated name of the county followed by the year of occurrence, and a chronological number if needed (i.e., AB/2020, B/2018, DB/2017, HR/2017, HR/2018, IS/2016, IS/2017_1, IS/2017_2, IS/2017_3, IS/2018, IS/2019, MM/2018, MM/2019_1, MM/2019_2, PH/2018, and VS/2018).

### 4.2. Multiple-Locus Variable-Number of Tandem Repeats Analysis (MLVA)

MLVA was performed as described in the standard operating procedure recommended by ECDC [14], which is based on the 5-locus MLVA method published by Hopkins et al. [15]. MLVA data were used to generate a minimum spanning tree (MST) in BioNumerics version 6.6 software (Applied Maths, Sint-Martens-Latem, Belgium) with the priority rule set so that the type which had the highest number of single-locus variants (SLVs) would be linked first. MLVA genotypes were regarded as MLVA clusters using one allele difference between 2 neighbouring MLVA genotypes as criterion.

### 4.3. Whole Genome Sequencing

The whole genomes of 20 *S*. Enteritidis strains, representative for three MLVA genotypes commonly assigned to the Romanian isolates and with epidemiological relevance at the European level as well, were sequenced on the Ion Torrent PGM platform (Thermo Fischer Scientific, Waltham, MA, United States). The DNA libraries were prepared using the 400-base read length chemistry. Enzymatic shearing of DNA samples and generation of barcoded libraries were carried out by using the Ion Xpress Plus Fragment Library Kit and Ion Xpress Barcode Adapters Kit (Thermo Fisher Scientific). Libraries were size-selected by electrophoresis using E-gel SizeSelect II Agarose Gel 2% (Thermo Fisher Scientific) and templates were prepared by using the Ion PGM Hi-Q View OT2 Kit and the Ion OneTouch 2 system (Thermo Fisher Scientific). Sequencing was conducted on Ion 318 chip v2 BC using the Ion PGM Hi-Q View Sequencing Kit (Thermo Fisher Scientific). For the assembly of sequences, SPAdes version 3.1.0, included in Torrent Suite 5.12.1. (Thermo Fisher Scientific, Waltham, MA, United States) was used, with default parameters (k-mers length set at 21, 33, 55, 77, and 99).

Multilocus sequence typing (MLST) profiles and core-genome MLST (cgMLST) alleles were generated from the fasta sequence data with SeqSphere+ software version 7.0 (Ridom GmbH, Münster, Germany) (accessed on 10 November 2021) using *S. enterica* MLST ver. 1.0 (Center for Genomic Epidemiology, Lyngby, Denmark) and *S. enterica* cgMLST ver. 2.0 schemes [16]. MST based on the cgMLST allelic profiles was constructed using SeqSphere^+^, with the option “pairwise ignoring missing values” turned on, and the default single-linkage threshold of ≤ 7 alleles maximum distance in cluster.

Additionally, single-nucleotide polymorphism (SNP)-based analysis was performed with Call SNPs and Infer Phylogeny (CSI) v. 1.4. tool freely available at the Center for Genomic Epidemiology’s website (www.genomicepidemiology.org (accessed on 12 June 2021) using the *S*. Enteritidis accession number AM933172 as a reference for the analysis and default parameters [17]. ResFinder 4.1 software also available at the Center for Genomic Epidemiology’s website and Basic Local Alignment Search Tool (BLAST) available at the National Center for Biotechnology Information (NCBI) website (https://blast.ncbi.nlm.nih.gov/Blast.cgi) (accessed on 13 June 2021) were used to detect acquired antimicrobial resistance genes and/or chromosomal mutations mediating antimicrobial resistance [18,19].

High throughput sequencing data of 12 *S*. Enteritidis strains isolated in other European regions available in European Nucleotide Archive (ENA) and EnteroBase (https://enterobase.warwick.ac.uk/species/index/senterica) (accessed on 10 May 2021) were also added to the data analyses. For gene-by-gene allele calling comparisons, the fasta sequences downloaded from EnteroBase were directly imported in SeqSphere+, while the raw sequence reads downloaded from ENA were first assembled de novo using SPAdes version 3.12.0 (used k-mers sizes: 21, 33, and 55) (Center for Algorithmic Biotechnology, St Petersburg, Russia,). The selection of the international strains was oriented by information from previously published studies and ECDC/EFSA reports referring to a large multi-country outbreak [5,20,21]. Of the genomes retrieved from the sequence databases to be compared with the Romanian strains, with the exception of four index strains used by ECDC/EFSA to delineate this international outbreak, only the strains that grouped within a difference threshold of 10 loci were kept for graphical representation.

In addition, the SPAdes assemblies with at least 95% core genome alleles [16] were compared against sequence data available in The European Surveillance System (TESSy) (https://www.ecdc.europa.eu/en/publications-data/european-surveillance-system-tessy (accessed on 30 March 2021)) to find matching isolates from other European countries differing in 7 core genome alleles or less. The data from TESSy is not included in the figures or tables of this study. This analysis was done only to screen for possible multi-country spread of the strains detected in Romania.

### 4.4. In Vitro Antimicrobial Susceptibility Testing

The susceptibility to antibiotics was determined by the disk diffusion method. The European Committee on Antimicrobial Susceptibility Testing (EUCAST) criteria were used for ampicillin, cefotaxime, ceftazidime, meropenem, nalidixic acid, ciprofloxacin, pefloxacin, gentamicin, trimethoprim, and trimethoprim, plus sulfamethoxazole, chloramphenicol, and the Clinical and Laboratory Standards Institute’s (CLSI) criteria for kanamycin, streptomycin, sulphonamides, and tetracycline. Resistance to ciprofloxacin was also verified by Etest (bioMérieux, Marcy-l’Étoile, France) [22,23].

### 4.5. Statistical Analysis

The discriminatory power of MLVA was evaluated in the 245 *S.* Enteritidis strains sampled with Simpson’s index of diversity (DI) using the formula:

DI = 1 − [Σ *n*(*n* − 1)/N(N − 1)] (1)

where *n* is the number of isolates belonging to a single type and N is the total number of isolates [24]. A total of 95% confidence interval (CI) was also calculated, as described previously [25].

## Figures and Tables

**Table 1:** MLVA types in association with the county distribution over the studied period and antibiotic susceptibility.

MLVA Genotype/ Cluster	Allelic Profile	No. of Strains	County Distribution ^1^					Phenotypic Antibiotic Susceptibility Profiles ^2^(No. of Strains)
			2016	2017	2018	2019	2020	
G1/CI	2-11-7-3-2	120	BZ, CV, DB, IS *^,3^	AB, BR, BT, BZ, CL, CT, CV, DB, HR *, IS *, OT, VS	B *, BR, BT, BV, BZ, DB, HR *, IS *, MH, OT, TL, VN	B, BT, BZ, DB, MH, MM *, OT, TM	AB *	Susceptible (83) S3 ^R^ (1) NA ^R^CIP ^R^PEF ^R^ (35) NA ^R^CIP^R^PEF ^R^S3 ^R^ (1)
G2/CI	2-10-7-3-2	30	IS, VN	BT, CV	BZ, MH, MM *, SV	B, BR, BZ, IS, MH, MM, TM		Susceptible (29) S3 ^R^ (1)
G3/CI	2-12-7-3-2	27		BN, BR, BZ, CV, DB, IS *	B, BV, BZ, DB, PH *	B, BZ, DB		Susceptible (4) NA ^R^CIP ^R^PEF ^R^ (23)
G4/CII	2-10-12-5-1	14			VS∗			Susceptible (14)
G5/CI	2-11-8-3-2	9	IS	CV, DB	BZ, DB	BR, DB		Susceptible (6) NA ^R^CIP ^R^PEF ^R^ (3)
G6/CI	2-9-7-3-2	9	IS	BZ, CV, DB	BZ, CV, SM	DB		Susceptible (8) AMP ^R^ (1)
G7	3-10-5-4-1	5	DB, MH			MM *		Susceptible (5)
G8/CI	2-11-6-3-2	4		DB *	CV, DB			Susceptible (2) S ^R^ (1) AMP ^R^NA ^R^CIP ^R^PEF ^R^ (1)
G9/CI	2-13-7-3-2	4	IS					Susceptible (4)
G10/CI	2-10-6-3-2	2			BR, DB			Susceptible (2)
G11/CI	2-12-9-3-2	2				BZ	B	Susceptible (1) NA ^R^CIP ^R^PEF ^R^ (1)
G12/CIII	3-11-5-3-1	2		DB	CL			Susceptible (1) NA ^R^CIP ^R^PEF ^R^ (1)
G13/CIII	3-11-5-4-1	2		BT	BT			Susceptible (2)
G14/CIII	3-9-5-4-1	2	MH	MM				Susceptible (1) NA ^R^CIP ^R^PEF ^R^ (1)
G15/CI	2-10-4-3-2	1				B		NA ^R^ (1)
G16/CI	2-10-8-3-2	1			SV			Susceptible (1)
G17/CI	2-10-8-5-2	1		DB				Susceptible (1)
G18	2-10-9-6-1	1		DB				Susceptible (1)
G19/CII	2-11-12-5-1	1			VS			Susceptible (1)
G20/CII	2-11-14-5-1	1				B		Susceptible (1)
G21/CI	2-11-7-4-2	1				DB		Susceptible (1)
G22/CI	2-11-8-5-2	1					B	Susceptible (1)
G23	2-12-6-3-2	1		CV				Susceptible (1)
G24/CII	2-14-11-5-1	1			DB			NA ^R^ (1)
G25	2-15-11-4-1	1				B		Susceptible (1)
G26/CI	2-9-8-3-2	1			BV			Susceptible (1)
G27/CI	2-9-8-4-2	1				BT		Susceptible (1)

^1^ County abbreviation: AB = Alba, BN = Bistriţa-Năsăud, BT = Botoşani, BR = Brăila, BV = Braşov, B = Bucureşti, BZ = Buzău, CL = Călăraşi, CT = Constanţa, CV = Covasna, DB = Dâmboviţa, HR = Harghita, IS = Iaşi, MM = Maramureş, MH = Mehedinţi, OT = Olt, PH = Prahova, SM = Satu Mare, SV = Suceava, TM = Timiş, TL = Tulcea, VS = Vaslui, VN = Vrancea; ^2^ AMP = ampicillin, CIP = ciprofloxacin, NA = nalidixic acid, PEF = pefloxacin, S = streptomycin, S3 = sulfonamides, R= resistant. ^3^ Asterisk indicates the geographic origin of outbreak-related strains.

## Data Availability

Raw reads sequences that support the findings of this study have been deposited in European Nucleotide Archive (ENA) at EMBL-EBI with the project accession code PRJEB40400 (ERS5088393–ERS5088412).

## Abbreviations

AB	Alba
BN	Bistriţa-Năsăud
BT	Botoşani
BR	Brăila
BV	Braşov
B	Bucharest
BZ	Buzău
CL	Călăraşi
CT	Constanţa
CV	Covasna
DB	Dâmboviţa
HR	Harghita
IS	Iaşi
MM	Maramures
MH	Mehedinţi
OT	Olt
PH	Prahova
SM	Satu Mare
SV	Suceava
TM	Timiş
TL	Tulcea
VS	Vaslui
VN	Vrancea
AMP	Ampicillin
CIP	Ciprofloxacin
NA	Nalidixic acid
PEF	Pefloxacin
S	Streptomycin
S3	Sulphonamides
R	Resistant
